# Risk Factors for Congenital Cryptorchidism in a Prospective Birth Cohort Study

**DOI:** 10.1371/journal.pone.0003051

**Published:** 2008-08-25

**Authors:** Ida N. Damgaard, Tina K. Jensen, The Nordic Cryptorchidism Study Group, Jørgen H. Petersen, Niels E. Skakkebæk, Jorma Toppari, Katharina M. Main

**Affiliations:** 1 University Department of Growth and Reproduction, GR-5064 Rigshospitalet, Copenhagen, Denmark; 2 Department of Physiology and Pediatrics, University of Turku, Turku, Finland; 3 Department of Biostatistics, University of Copenhagen, Copenhagen, Denmark; JARING, Malaysia

## Abstract

**Background:**

Risk factors for congenital cryptorchidism were investigated in a prospective birth cohort study in Denmark and Finland from 1997 to 2001.

**Methodology and Principal Findings:**

In total, 2,496 boys were examined for cryptorchidism at birth (cryptorchid/healthy: 128/2,368) and three months old (33/2,215). Information on risk factors was obtained antenatally (questionnaire/interview) or at birth from birth records. Use of nicotine substitutes during pregnancy (*n* = 40) and infertility treatment by intrauterine insemination (*n* = 49) were associated with an increased risk for cryptorchidism, adjusted odds ratio (95% confidence interval) (OR (95%CI)) 3.04 (95%CI 1.00–9.27) and 3.01 (95%CI 1.27–7.15), respectively. No association was seen for mothers (*n* = 79) who had infertility treatment in form of intracytoplasmic sperm injection (ICSI) or in vitro fertilization (IVF) treatment (OR 0.71 95%CI 0.21–2.38). In total, 728 (29%) reported to have smoked during pregnancy, however, no increased risk among maternal smokers was found. Furthermore, we found statistically significant associations between cryptorchidism and low birth weight, prematurity, being small for gestational age, substantial vaginal bleeding, and breech presentation, which are in accordance with other studies.

**Conclusions and Significance:**

Our study revealed two novel risk factors for cryptorchidism: intrauterine insemination and the use of nicotine substitutes in pregnancy. This suggests that cryptorchidism may not only be associated to genetic factors, but also to maternal lifestyle and exposure.

## Introduction

Cryptorchidism is the most common congenital genital malformation in males. In the Western countries, the condition is estimated to occur in 2–5% of full-term newborn boys [1]. Most cases appear isolated without other malformations, and only very few are part of genetic or endocrine syndromes. Cryptorchidism is the best described risk factor for testicular cancer [2], [3]. In some cases, defects in specific genes, e.g. *INSL3* seem to be involved [4], but although a variety of risk factors have been reported, e.g. low birth weight, prematurity, low parity and twinning [5]–[8], the aetiology remains largely unknown. Several studies indicate an increase in the prevalence of congenital cryptorchidism within a few generations supporting the hypothesis that lifestyle changes and environmental factors may be involved [9]–[12].

Normal testicular development and descent in humans is strongly dependent on normal sex hormone balance [1] and may thus be sensitive to adverse environmental and lifestyle exposures not only in the first trimester but throughout the entire pregnancy.

Most previous studies of risk factors for cryptorchidism have included data obtained retrospectively by either self-administered questionnaires or interviews conducted post partum [13]–[19]. These studies may therefore be compromised by recall and selection bias. Furthermore, several studies have been based on registry data, which may be hampered by variations in ascertainment of the diagnosis and reporting strategies [20]. To overcome this, we conducted a prospective, population-based cohort study of pregnant women and their newborn boys, in which information on risk factors was obtained antenatally and from birth records at birth and in which all included boys were examined under standardised conditions in order to ensure a valid diagnosis.

We have previously described an association between maternal alcohol consumption during pregnancy and cryptorchidism in this cohort [21]. We have earlier also shown an association of an increased risk of cryptorchidism to low birth weight and prematurity [10]. In this study, we investigated additional infant, maternal and delivery characteristics associated with cryptorchidism.

## Methods

We performed a prospective birth cohort study at the University Hospital of Copenhagen (Rigshospitalet and Hvidovre Hospital) in Denmark from 1997 to 2001 and at the Turku University Central Hospital in Finland in the period 1997–99. Eligible women (2,229 Danish and 2,728 Finnish) residing in the hospital referral areas were consecutively recruited during early pregnancy. In Denmark, eligible families (selected on Danish surname) were contacted by mail. In Finland, the women were recruited on their first visit at the antenatal clinics. Women referred from outside the recruitment area because of pregnancy complications were excluded. In order to obtain genetically and environmentally well-defined populations, only families who met the following criteria were included: both parents and grandparents of the unborn child had to be born and raised in Denmark, or respectively in Finland. A maximum residence abroad of three years for the mother and ten years for the father and grandparents was allowed.

Of 4,957 women initially included 2,639 gave birth to 2,666 boys. A total of 95 families (including 5 pairs of boy–boy twins) dropped out before the first examination (4 moved, 44 uninterested, 3 child dead, 7 child sickness, 30 lost, 7 other reasons). Furthermore, 58 boys were not examined at birth, 8 were not included due to a missing questionnaire and 4 were excluded for other reasons (1 unilateral torsion, 1 unilateral agenesis, 2 could not be classified at birth due to severe bilateral hernia) [21].

The study was conducted in two different countries and therefore questionnaires, interviews and examinations were strictly standardized. The families were included in early pregnancy after written informed consent. The participating women received a self-administered questionnaire late in 1st trimester or early in 2nd trimester covering education and occupation, maternal pre-pregnancy weight and height, medical and obstetric history. All women were asked about infertility treatment (yes/no) and if yes, which type (insemination, ICSI/IVF). They were also asked whether the semen was husband or donor provided and if they had experienced any pregnancy-related complications, e.g. bleeding during pregnancy (duration and extent) and pre-eclampsia or pregnancy-induced hypertension. The intensity of vaginal bleeding was classified by duration (≤1 day/>1day) and extent (spot bleeding/more than spot bleeding). Time to pregnancy was defined at time period from cessation of use of anti-conception. Furthermore, they were asked about daily smoking habits (type, amount, duration) and changes in habits during pregnancy (quit smoking (gestational week), reduced smoking (gestational week), occasional smoking) and use of substitutes in form of nicotine patches, spray or chewing gum (duration, trimester) and exposure to passive smoking (daily extent). Based on these questions the women were categorized into different smoking categories (nonsmoker, current smoker, stop of smoking before pregnancy/during pregnancy and occasional smoker). Nonsmokers were defined as women who had never been smoking in their entire life. Information on other maternal lifestyle factors such as daily caffeine intake (cups of coffee and tea) and weekly alcohol intake [21] were also obtained. The women also reported self-experienced stress (yes/no). They were instructed to complete the questionnaire at the beginning of the 3rd trimester and to return it before birth. Data on pregnancy-related complications such as pre-eclampsia and bleeding during pregnancy were also obtained from hospitals records at birth.

Social class was determined from self-reported occupational status of the mother in seven hierarchical categories: higher-grade professionals, lower-grade professionals, skilled workers, unskilled workers, students, economically inactive and unclassifiable [22].

Simultanenously to the present cohort, but independent of it, a national birth cohort study was conducted [Danish National Birth Cohort (DNBC)] in Denmark [23]. The women participating in DNBC answered two telephone interviews at around pregnancy week 12 and 30, which contained most of the questions asked in the self-adminstrered questionnaire used in our cohort. The interviews and the questionnaire were developed in close collaboration and therefore almost identical. In total, 495 Danish women participated both in our study and in DNBC. Of these 210 women completed a shortened questionnaire to avoid unnecessary repetition. The other 285 completed the entire questionnaire and both interviews.

The study was conducted according to the Helsinki II Declaration (http:/www.wma.net/e/policy/b3.htm) [24] and was approved by the local ethical committees in both countries (Finland: 7/1996, Denmark: KF01-030/97) and the Danish Data Protection Agency (1997-1200-074, 2001-3311-0068).

### Clinical examination

The boys were examined at birth and 3 months old. Preterm born boys were examined at the expected date of delivery. Gestational age was based on routine ultrasonography performed in pregnancy week 18–20. If not available (2.1%), the last menstrual period was used. Information on birth weight and parity was obtained from birth records. Weight for gestational age (WGA) was calculated according to national standards [25]–[27]. Repetitive workshops were held in order to ensure standardization of the clinical procedures and to minimize inter-observer variation. One Finnish doctor spent 1 year in Denmark allowing a close harmonization of the examination techniques.

Definition of cryptorchidism and examination technique have been described earlier [10]. The testis was considered cryptorchid, if found in a high scrotal, supra-scrotal or inguinal position or if it was non-palpable. Retractile testis was considered to be a normal variant. The results presented in Tables 1–3 are based on the diagnosis at the newborn examination, without sub-division to cryptorchidism types.

**Table 1:** Population characteristics of 128 boys with congenital cryptorchidism and 2,368 healthy boys from a joint Danish-Finnish birth cohort study.

Variable	Subvariable	Cryptorchid	Healthy	OR (95%CI)	OR (95%CI)
		*n* = 128	*n* = 2,368	Unadjusted	Adjusted*
Hypospadias		2 (1.6)	10 (0.4)	3.74 (0.81–17.26)	1.08 (0.12–9.44)
Twinning		7 (5.5)	58 (2.4)	2.30 (1.03–5.16)	0.52 (0.19–1.47)
Birth weight (grams)	<2500	14 (10.9)	54 (2.3)	3.78 (1.98–7.20)	2.73 (1.14–6.55)
	2500–3500	58 (45.3)	845 (35.7)	1 (ref)	1 (ref)
	>3500	56 (43.8)	1,469 (62.0)	0.56 (0.38–0.81)	0.67 (0.44–1.04)
Maturity	premature (<37 weeks)	18 (14.1)	103 (4.3)	3.58 (2.09–6.13)	2.14 (1.03–4.46)
	mature (37–42 weeks)	105 (82.0)	2,152 (90.9)	1 (ref)	1 (ref)
	postmature (>42 weeks)	5 (3.9)	113 (4.8)	0.91 (0.36–2.27)	0.99 (0.38–2.56)
Weight for gestational age (WGA)**	SGA	8 (6.3)	57 (2.4)	2.75 (1.28–5.89)	2.55 (1.06–6.13)
	AGA	115 (89.8)	2,251 (95.1)	1 (ref)	1 (ref)
	LGA	5 (3.9)	60 (2.5)	1.63 (0.64–4.14)	2.04 (0.77–5.39)
Parity	1	79 (61.7)	1,369 (57.8)	1 (ref)	1 (ref)
	2	34 (26.6)	700 (29.6)	0.84 (0.56–1.27)	1.15 (0.71–1.85)
	≥3	15 (11.7)	299 (12.6)	0.87 (0.49–1.53)	1.28 (0.64–2.59)
Presentation	Head	115 (90.6)	2,277 (96.4)	1 (ref)	1 (ref)
	Breech	12 (9.4)	71 (3.0)	3.35 (1.77–6.35)	2.59 (1.12–5.97)
	Other	0	14 (0.6)	-	-
Delivery	Vaginal	83 (64.8)	1,787 (75.5)	1 (ref)	1 (ref)
	Vacuum	16 (12.5)	239 (10.1)	1.44 (0.83–2.50)	1.54 (0.84–2.83)
	Caesarean section	29 (22.7)	342 (14.4)	1.83 (1.18–2.83)	1.04 (0.57–1.89)

Values are given as numbers (*n*) and percentages (%).

* All variables adjusted for country, maternal age, smoking, alcohol consumption and social class. Furthermore, adjusted for parity, twinning, mode of delivery, presentation, birth weight (except WGA) and gestational age (except WGA), mutually.

** SGA: small for gestational age, AGA: appropriate for gestational age, LGA: large for gestational age.

**Table 2 pone-0003051-t002:** Parental characteristics of 128 boys with congenital cryptorchidism and 2,368 healthy boys in a joint Danish-Finnish birth cohort study.

	Cryptorchid	Healthy	OR (95%CI)	OR (95%CI)
	*n* = 128	*n* = 2,368	Unadjusted	Adjusted*
Maternal pre-pregnancy BMI				
<20	20 (16.3)	398 (17.1)	0.97 (0.58–1.60)	0.91 (0.53–1.57)
20–25	71 (57.7)	1,363 (58.7)	1 (ref)	1 (ref)
>25	32 (26.0)	561 (24.2)	1.10 (0.71–1.68)	1.14 (0.72–1.80)
Maternal social class				
1+2:higher and low grade professionals	46 (39.0)	808 (36.7)	1 (ref)	1 (ref)
3+4:skilled and unskilled workers	51 (43.2)	1,071 (48.7)	0.84 (0.56–1.26)	1.00 (0.66–1.52)
5+6:students and economically inactive	21 (17.8)	322 (14.6)	1.15 (0.67–1.95)	1.15 (0.67–1.96)
Maternal age (years)				
<30	62 (48.4)	1,231 (52.0)	1 (ref)	1 (ref)
≥30	66 (51.6)	1,137 (48.0)	1.15 (0.81–1.65)	0.95 (0.64–1.40)
Paternal age (years)				
<30	35 (33.3)	846 (39.3)	1 (ref)	1 (ref)
≥30	70 (66.7)	1,307 (60.7)	1.30 (0.86–1.96)	1.05 (0.68–1.62)
Maternal smoking				
Smoking categories				
0: nonsmoker**	58 (45.7)	1,172 (49.5)	1 (ref)	1 (ref)***
1: cessation before pregnancy	31 (24.4)	506 (21.4)	1.24 (0.79–1.94)	1.05 (0.61–1.80)
2: occasional smoker	1 (0.8)	44 (1.9)	0.46 (0.06–3.39)	-
3: cessation during pregnancy	19 (15.0)	360 (15.2)	1.07 (0.63–1.81)	0.87 (0.43–1.76)
4: current smoker	18 (14.2)	286 (12.1)	1.27 (0.74–2.19)	0.90 (0.43–1.87)
Smoking substitute				
nonsmoker	58 (45.7)	1,172 (49.5)	1 (ref)	1 (ref)***
smoking categories 1–4−substitutes	63 (49.6)	1,162 (49.1)	1.10 (0.76–1.58)	0.84 (0.53–1.33)
smoking categories 1–4+substitutes	6 (4.7)	34 (1.4)	3.57 (1.44–8.83)	3.04 (1.00–9.27)

Values are given as numbers (*n*) and percentages (%).

* Adjusted for country and social class.

** Nonsmoker: woman who has never been smoking in her entire life.

*** Adjusted for country, social class, birth weight, stress, alcohol and caffeine intake.

**Table 3:** Number of planned pregnancies, time to pregnancy, infertility treatment and pregnancy related complications in a joint Danish-Finnish birth cohort study.

Variable	Subvariable	Cryptorchid	Healthy	OR (95%CI)	OR (95%CI)
		*n* = 128	*n* = 2,368	Unadjusted	Adjusted*
Pregnancy planned	yes/partly	111 (88.8)	2,119 (90.4)	1 (ref)	1 (ref)
Pregnancy planned	no	14 (11.2)	225 (9.6)	1.19 (0.67–2.11)	0.90 (0.49–1.67)
Time to pregnancy**	<4	65 (72.2)	1,206 (63.1)	1 (ref)	1 (ref)
Time to pregnancy**	4–12	12 (13.3)	384 (20.1)	0.58 (0.31–1.09)	0.60 (0.32–1.14)
	>12	13 (14.4)	320 (16.8)	0.75 (0.41–1.39)	0.70 (0.36–1.35)
Infertility treatment	none	102 (91.1)	2,021 (94.5)	1 (ref)	1 (ref)
Infertility treatment	insemination	7 (6.3)	42 (2.0)	3.30 (1.45–7.53)	3.01 (1.27–7.15)
	ICSI/IVF	3 (2.7)	76 (3.6)	0.78 (0.24–2.52)	0.71 (0.21–2.38)
Nausea	no	45 (36.9)	802 (34.6)	1 (ref)	1 (ref)
Nausea	yes	77 (63.1)	1,517 (65.4)	0.91 (0.62–1.32)	0.87 (0.58–1.30)
Vomiting	no	78 (72.2)	1,473 (66.8)	1 (ref)	1 (ref)
Vomiting	yes	30 (27.8)	733 (33.2)	0.77 (0.50–1.19)	0.81 (0.52–1.26)
Pre-eclampsia or hypertension	no	84 (90.3)	1,933 (95.3)	1 (ref)	1 (ref)
Pre-eclampsia or hypertension	yes	9 (9.7)	95 (4.7)	2.18 (1.06–4.47)	1.72 (0.79–3.75)
Substantial bleeding	no	118 (92.9)	2,269 (97.0)	1 (ref)	1 (ref)
Substantial bleeding	yes	9 (7.1)	69 (3.0)	2.51 (1.22–5.15)	2.23 (1.06–4.69)

Values are given as numbers (*n*) and percents (%).

* Adjusted for country, maternal age, social class and parity.

** Time to pregnancy from cessation use of anti-conception.

### Statistical analysis

Gestational age for completing questionnaires and interviews is given as mean (standard deviation (SD)) and differences tested by unpaired T-tests. Descriptive data of the mothers and the boys are given as numbers and percentages (%). Differences between cryptorchid boys and healthy boys were tested by Fischer's exact test (2-sided) or described by odds ratios (OR) and 95% confidence intervals (95%CI). Odd ratios (unadjusted and adjusted) were estimated using binary logistic regression. The covariates, which were most closely associated with the investigated exposure and cryptorchidism and factors which due to a priori knowledge from other studies that they may influence the outcome, were included as confounders in the analyses.

### Validation study

Concerning smoking habits, a validation of comparability between questionnaire and interview data, calculated in percent of agreement, was performed among 285 Danish women, who participated in both. Complete agreement in the answers concerning smoking categories was found for 265 women (93%). Minor disagreement was registered for 18 women (6%); 12 (4%) women reported to have smoked occasionally during pregnancy in the questionnaire but were classified at the interview as nonsmokers (*n* = 7) or having stopped during pregnancy (*n* = 5). Six women (2%) who had quit smoking in early pregnancy (questionnaire) were categorized as nonsmokers in the interview. Major disagreement was found for 2 women (0.7%). One reported smoking cessation before pregnancy in the questionnaire, but answered to have stopped smoking while pregnant in the interviews. One reported to be a smoker in the questionnaire, but was registered as a nonsmoker in the interviews. The 285 women were included in the study with their questionnaire data.

## Results

In Denmark, 1,042 boys (1,029 mothers) and in Finland 1,454 boys (1,446 mothers) participated in the study [21]. In total, 128 boys (94 Danish, 34 Finnish) were cryptorchid at birth, 33 (19 Danish, 14 Finnish) remained cryptorchid at the age of 3 months.

All interviews were conducted antenatally. Most questionnaires (94%) were returned before birth. Return date was missing for 104 women [12 (9.4%) mothers of cryptorchid boys and 92 (3.9%) mothers of normal boys, *p* = 0.002]. Two mothers of cryptorchid boys and 37 mothers of normal boys completed the questionnaire after birth (*p* = 0.687). Mean gestational age for completing of the questionnaire/second interview for mothers of cryptorchid boys was 199 days [standard deviation (SD) 41] versus 207 days (58) for mothers of normal boys (*p* = 0.211). The corresponding figures for participating in the first interview were 113 days (33) and 115 days (30), *p* = 0.755.

Table 1 shows population characteristics. Cryptorchid boys had an increased risk of hypospadias and twinning [odds ratios (OR) 3.74 and 2.30, respectively], however these differences were not significant after adjustment for confounders. Low birth weight, prematurity and being small for gestational age (SGA) were all statistically significantly associated to cryptorchidism, also after adjusting for confounders (OR 2.73, 2.14 and 2.55, respectively). In total, 12 (9.4%) cryptorchid boys and 71 (3.0%) healthy boys presented as breech at birth, a difference that remained significant after adjustment. Stratified analyses showed that this difference was statistically significant among mature boys (premature: OR 4.03 95%CI 0.71–22.94, mature OR 3.44 95%CI 1.46–8.13). Caesarean section was more common among cryptorchid boys (OR 1.83 95%CI 1.18–2.83), however, this difference did not remain significant after adjusting. The odds ratios of cryptorchidism according to parental characteristics are given in Table 2. No significant differences were seen for maternal pre-pregnancy body mass index (BMI). No effect of parental age was observed.

No increased risk of cryptorchidism among the offspring of smokers was found (Table 2). Exposure to passive smoking had no effect either (data not shown). Smoking mothers of cryptorchid boys did not smoke significantly more cigarettes than smoking mothers of healthy boys (*n* = 294, mean 6.9 versus 5.8, *p* = 0.458). Heavy smokers, i.e. women smoking ≥10 cigarettes/day (*n* = 64), did not show an increased risk for having a cryptorchid boy compared to nonsmokers (adjusted OR 0.59 95%CI 0.16–2.24). Heavy smokers did not have significantly more boys with bilateral cryptorchidism compared to nonsmokers (33.3% versus 24.1%, *p* = 0.635); 17% (*n* = 63) of the women who stopped smoking while pregnant (*n* = 379) reported to have smoked ≥10 prior to cessation. Including these women in the analyses as heavy smokers did not change the results (data not shown). Smoking cessation was most common in the first trimester (*n* = 323, 85%), whereas 12% and 3% reported to have stopped during the 2nd and 3rd trimester, respectively.

Users of nicotine substitutes (*n* = 40) (disregarding their current smoking habits) had an increased risk of cryptorchidism in their offspring (OR 3.04 95%CI 1.00–9.27). Both women who ceased smoking during pregnancy (*n* = 20) and used substitutes (OR 4.60 95%CI 1.10–19.23), and women who continued smoking throughout the entire pregnancy (*n = *16) and used substitutes had increased odds (OR 2.42, 95%CI 0.46–12.70), albeit statistically significance was not reached for the latter. Four women who stopped smoking before pregnancy used nicotine substitutes during pregnancy (all healthy sons).

Compared to nonsmokers, substitute users were older, more often reported to have been dis-stressed during pregnancy, belonging to social class 1 or 2 and had a smaller weekly alcohol intake, a higher daily intake of tea and coffee, whereas no differences were seen in birth weight, parity or gestational age (data not shown). Mothers who used nicotine substitutes did tend to smoke more cigarettes per day than women who did not use substitutes (mean 9.6 versus 6.2, respectively *p* = 0.010). Exclusion of heavy smokers (≥10 cigarettes per day) from the analyses did not change the results (data not shown). Information on type of nicotine substitute was available for 5 of 6 mothers giving birth to cryptorchid boys and for 30 out of 34 mothers giving birth to normal boys. Self-reported maternal stress during the last 6 months was associated with cryptorchidism (OR 1.79 95%CI 1.17–2.74), although the association was not significant after adjustment (OR 1.34 95%CI 0.85–2.12). Table 3 shows pregnancy-related complications. An increased risk for cryptorchidism was found among mothers who reported pre-eclampsia/pregnancy-induced hypertension or vaginal bleeding. After adjusting the difference remained statistically significant only for substantial vaginal bleeding (OR 2.23, 95%CI 1.06–4.69).

No difference in time to pregnancy was found between mothers to healthy and cryptorchid boys. Significantly more mothers to cryptorchid boys had been treated with intrauterine insemination (OR 3.01 95%CI 1.27–7.15). Semen originated from the father among 45 boys (7 cryptorchid sons, 38 healthy sons) and from a donor among 4 boys (all healthy). Mothers who had intrauterine insemination were older, had a lower parity, a longer waiting time to pregnancy (TTP) and more were from social class 1 or 2 than mothers who did not have any infertility treatment (data not shown). No association was seen for mothers who had infertility treatment in form of intracytoplasmic sperm injection (ICSI) or in vitro fertilization (IVF) treatment (OR 0.71, 95%CI 0.21–2.38).

Information on infertility treatment was missing for 16 (12.5%) mothers of cryptorchid boys and for 229 (9.7%) mothers of normal boys, *p* = 0.295. Including missing data concerning infertility treatment as having no infertility treatment did not change the results [adjusted OR (95%CI) for insemination and ICSI/IVF treatment 3.13 (1.32–7.41) and 0.75 (0.23–2.52), respectively].

Analysis of boys who remained cryptorchid at 3 months of age showed comparable results to boys with cryptorchidism at birth (data not shown), which however, did not reach statistical significance due to the small group size (*n* = 33).

## Discussion

In this prospective study of risk factors for cryptorchidism, we found that significantly more mothers of cryptorchid boys had been treated with intrauterine insemination and used nicotine substitutes than mothers of normal boys, which to our knowledge has not been described before. However, no increased risk among mothers treated with IVF/ICSI was observed.

No detailed information about the infertility treatment regime or the number of treatments was obtained. However, in both countries the majority of intrauterine insemination treatment will include clomiphene citrate administered orally, which exhibits both estrogenic and anti-estrogenic activity [28]. The half-life of clomiphene is up to 1 month, and it may accumulate over consecutive cycles of treatment [29]. It is therefore biologically plausible that the fetus could have been exposed to this compound during the critical stages of development in the first trimester. Animal studies have reported that clomiphene administration shortly after birth to male rats had long-term adverse effects on the reproductive physiology and sexual behaviour [30], [31]. Adverse effects such as epithelium changes on the developing female reproductive tract in humans exposed antenatally to tamoxifen or clomiphene, resembling those seen after prenatal diethylstilbestrol exposure, have also been reported [32]. The effects of clomiphene on the developing reproductive tract in human males have been reported in a few studies. Berkowitz et al found that clomiphene use before conception was associated with a 2-fold increase in risk of cryptorchidism, which however did not reach statistical significance [13]. One study indicated that clomiphene treatment of the mother was associated with penoscrotal hypospadias in the male offspring [33]. In addition, other exogenous hormonal treatments, especially diethylstilbestrol, but also hormonal contraception and hormonal pregnancy tests have previously been found to be associated with an increased risk of congenital malformations, including cryptorchidism [18], [34], [35], [36]–[41].

Surprisingly, we found no adverse effect of ICSI/IVF treatment on the risk of cryptorchidism. This may be explained by a different hormonal treatment regimen, consisting of initial suppression of the pituitary axis with a gonadotropin releasing hormone analogue, followed by follicle stimulating hormone, human menopausal gonadotropin and in some cases recombinant human luteinizing hormone. In general the half-life of these compounds is shorter than that of clomiphene. Previous registry studies of birth defects among children conceived after ICSI/IVF have found an increased risk for hypospadias, but not for cryptorchidism [42], [43].

As expected, mothers who had insemination treatment were older, had a lower parity, a longer waiting time to pregnancy (TTP) and more were from social class 1 or 2 than mothers who did not have infertility treatment. Thus, parental subfertility *per se* may be a risk factor for cryptorchidism. The demographic factors in couples treated with ICSI and IVF resemble those treated with insemination as to age, parity, TTP and social class, but these treatments are used in more severe cases of infertility and male factor infertility (ICSI). The percentages of mothers receiving infertility treatment in our cohort resemble those of the general Danish population [44]. We found no increased risk for cryptorchidism among subfertile women (with a long TTP) who did not receive treatment.

We did not find any association between maternal smoking and cryptorchidism, which is supported by most previous studies [7], [13], [16], [45], [46]. One recent study has, however, found that heavy maternal smoking (≥10 cigarettes/day, registered retrospectively) increased the risk of bilateral cryptorchidism [47]. In our study, heavy smokers did not have an increased risk of having a cryptorchid boy compared to nonsmokers and the distribution of bilateral versus unilateral cryptorchidism did not differ significantly either. However, we found that mothers who used nicotine substitutes had a significantly increased risk of having a cryptorchid son. A recent publication reported that children born to mothers who used nicotine substitutes during the first trimester had a slightly increased risk of congenital malformations, particularly musculoskeletal defects [48]. It should be noted that this study only examined the use of substitutes in the first 12 weeks of pregnancy in women who had stopped smoking before pregnancy. In our study both women who stopped smoking during pregnancy and women who continued smoking despite simultaneous use of substitutes were included. The group of women who stopped smoking before pregnancy but still used nicotine substitutes during pregnancy was too small (*n* = 4) to make separate analyses and the two studies are therefore not exactly comparable. We do not know if substitute users who quit smoking while pregnant, stopped smoking before or at the same time as they started to use substitutes or if they both smoked and used substitutes for a period. No information on precise amount or doses and the precise duration of the use of the substitutes was obtained. Women who used substitutes tended to smoke more than women who did not use substitutes, which would be expected. However, we do not believe that the effect seen of using substitutes could be explained by this difference, as comparable results were found when heavy smokers were excluded from the analyses. Obvious confounders such as alcohol and coffee/tea consumption did not affect our results, but we cannot exclude the association to be confounded by other lifestyle factors not analyzed in our study, e.g. diet. Our findings need to be confirmed in larger groups of women who smoke and use nicotine substitutes. It may not be nicotine *per se* that constitutes the risk factor, but other chemical substances in the delivery devices that women are exposed to concomitantly.

In accordance with many previous studies low birth weight, prematurity and being small for gestational age were independent risk factors of cryptorchidism [8], [12], [13], [46]. As in some previous studies [19], [39], [46], [49], we found associations between caesarean section, breech presentation and cryptorchidism. However, after adjusting caesarean section was no longer significant risk factor, which indicates that caesarean section reflects other risk factors such as prematurity, low birth weight and/or abnormal presentation. Although breech delivery *per se* has been shown to be associated with testicular damage, it is unlikely that the observed association is due to mechanical factors alone, as most of these boys in our study were delivered by cesarean section. Breech presentation may therefore be a marker for a maternal or fetal characteristics associated with cryptorchidism, e.g. placental impairment such as placenta praevia or low placenta weight [50].

Mothers with substantial bleeding during pregnancy had a significantly increased risk of having a cryptorchid boy. Vaginal bleeding may be an indicator of placenta malfunction, which in turn, may affect human choriogonadotropin (hCG) production and stimulation of testicular hormonal synthesis. Previously published studies on vaginal bleeding show conflicting results [7], [13], [16], [18]. High maternal BMI, vomiting and nausea, low parity, twinning and young maternal age are risk factors considered to indicate high endogenous maternal estrogen levels [51], but previous data are not consistent. [6], [7], [17], [49]. We were not able to detect any associations to these risk factors, which may be caused by the fact that our prospective cohort included relatively few mothers who exhibited these risk factors. An apparent increased risk for twins disappeared after adjusting for relevant confounders, e.g. low birth weight, which is in accordance to other studies [8]. Only few mother had a high BMI and our study may therefore be too small to detect an adverse effect of maternal BMI.

We conducted our study as a prospective cohort, in which information on maternal exposure were collected before birth in order to minimise recall-bias, which strengthens our findings. Furthermore, all the children were examined by trained observers who participated in repetitive workshops in order to ensure a valid diagnosis. Most mothers completed the questionnaire in the 3rd trimester and the range of gestational age for completing the questionnaire did not differ between cryptorchid and normal boys. Only 22–24% of all eligible women participated in our study and mothers with an academic degree were over-represented. Our findings are therefore not representative of the entire Danish and Finnish population. However, this should not bias our estimates of the association between potential risk factors and cryptorchidism, as the data was collected prospectively and therefore any misclassification of exposure and outcome was most likely to be non-differential, causing a bias toward the null hypothesis. Furthermore, the cohort design also minimized the risk of selection bias among the healthy boys. Finally, in Finland, the entire hospital birth cohort was followed in the same period, and its cryptorchidism prevalence was similar to that of this prospective cohort, suggesting that there was no selection bias [10]. The limitation of the prospective design is the relative small number of cases resulting in many non-significant results after adjustment for confounders.

Validation revealed a very good agreement in reporting smoking habits by interview and questionnaire, and we do not believe that this may have introduced bias. Furthermore, sensitivity analyses including missing values did not change the overall results. The study examined association with many risk factors, and some of the reported findings may therefore be spurious due to multiple testing. However, the association with several previously reported risk factors for cryptorchidism were confirmed.

In our study, most boys with congenital cryptorchidism showed spontaneous descent after birth. Thus, our group of cryptorchid boys is not directly comparable to studies including, e.g. children scheduled for orchidopexy, who have persistent cryptorchidism. We have previously shown, that these transient and usually mild cases of cryptorchidism also show signs of subtle impairment of testicular function at three months of age [52]. The geographical difference in the prevalence of cryptorchidism between Denmark and Finland and the increase in the Danish prevalence is mostly due to this type of cryptorchidism [10]. Thus, lifestyle factors may in particular be involved in the aetiology of mild types of cryptorchidism.

In conclusion, we found that intrauterine insemination and the use of nicotine substitutes were associated with an increased risk of having a boy with cryptorchidism. This suggests that maternal lifestyle factors and exposures play a role for testicular descent in humans. During the last decades infertility treatment has become an increasing part of the health care system [44]. Likewise, an increased public awareness for the adverse effects of smoking during pregnancy may encourage an increasing number of women to quit smoking, some of which with the aid of nicotine substitutes [53]. The above-mentioned factors may thus be contributing to the increased prevalence of congenital cryptorchidism, which warrants further studies.
